# Case Report: Experience in the diagnosis and treatment of a male patient with plasma cell mastitis in the lump stage and review of the literature

**DOI:** 10.3389/fmed.2025.1595042

**Published:** 2025-12-16

**Authors:** Li-Juan Chen, Zheng Dang

**Affiliations:** Department of Breast Surgery, 940 Hospital of the Joint Service Support Force of the Chinese People's Liberation Army, Lanzhou, China

**Keywords:** plasma cell mastitis, male, surgical treatment, case report, obesity, nipple inversion, diagnosis

## Abstract

Plasma cell mastitis (PCM) is a rare chronic inflammatory disorder primarily affecting non-lactating women, with fewer than 100 male cases reported globally. Male PCM is characterized by nonspecific clinical and radiological features that frequently mimic breast cancer, leading to diagnostic delays and mismanagement. Herein, we present a 55-year-old male patient with a 6-month history of a left subareolar mass initially misdiagnosed as malignancy due to spiculated margins on ultrasound (BI-RADS 4C) and mammography (BI-RADS 5). Despite negative percutaneous biopsy and normal tumor markers, surgical excision was performed following multidisciplinary evaluation. Pathological analysis confirmed PCM with dense plasma cell infiltration, multinucleated giant cells, and ductal ectasia. The patient achieved complete remission after margin-negative resection and remained recurrence-free at 12-month follow-up. Male PCM pathogenesis differs from female cases, with obesity (BMI 31 kg/m^2^) and nipple inversion identified as key risk factors in this case. Obesity likely contributes via adipose tissue-mediated estrogen dysregulation and hypoxia-induced inflammation, while nipple inversion promotes ductal obstruction and antigenic debris accumulation. This case highlights the diagnostic challenges of male breast masses, emphasizing the necessity of histopathological confirmation to differentiate PCM from carcinoma. Surgical excision remains the gold standard for localized disease, as nonsurgical approaches (e.g., antibiotics, glucocorticoids) often fail to prevent recurrence. Our findings underscore the importance of early surgical intervention in male PCM to optimize outcomes and reduce malignancy-associated morbidity. Further studies are needed to establish evidence-based guidelines for this understudied condition.

## 1 Introduction

Plasma cell mastitis (PCM) is a chronic non-infectious inflammatory disorder characterized by ductal ectasia and dense plasma cell infiltration, presenting clinically with breast pain, palpable masses, nipple discharge, abscess formation, and potential fistula/sinus tract development. It primarily affects non-lactating women aged 30–40 (1, 2), with a rare male incidence and delayed disease onset compared to other mastopathies. The etiology remains poorly understood, and radiological features often overlap with breast carcinoma, necessitating histopathological confirmation for diagnosis. While no universally accepted treatment protocol exists, surgical excision is frequently employed to expedite resolution and minimize recurrence risk.

## 2 Case description

**Figure 1:**
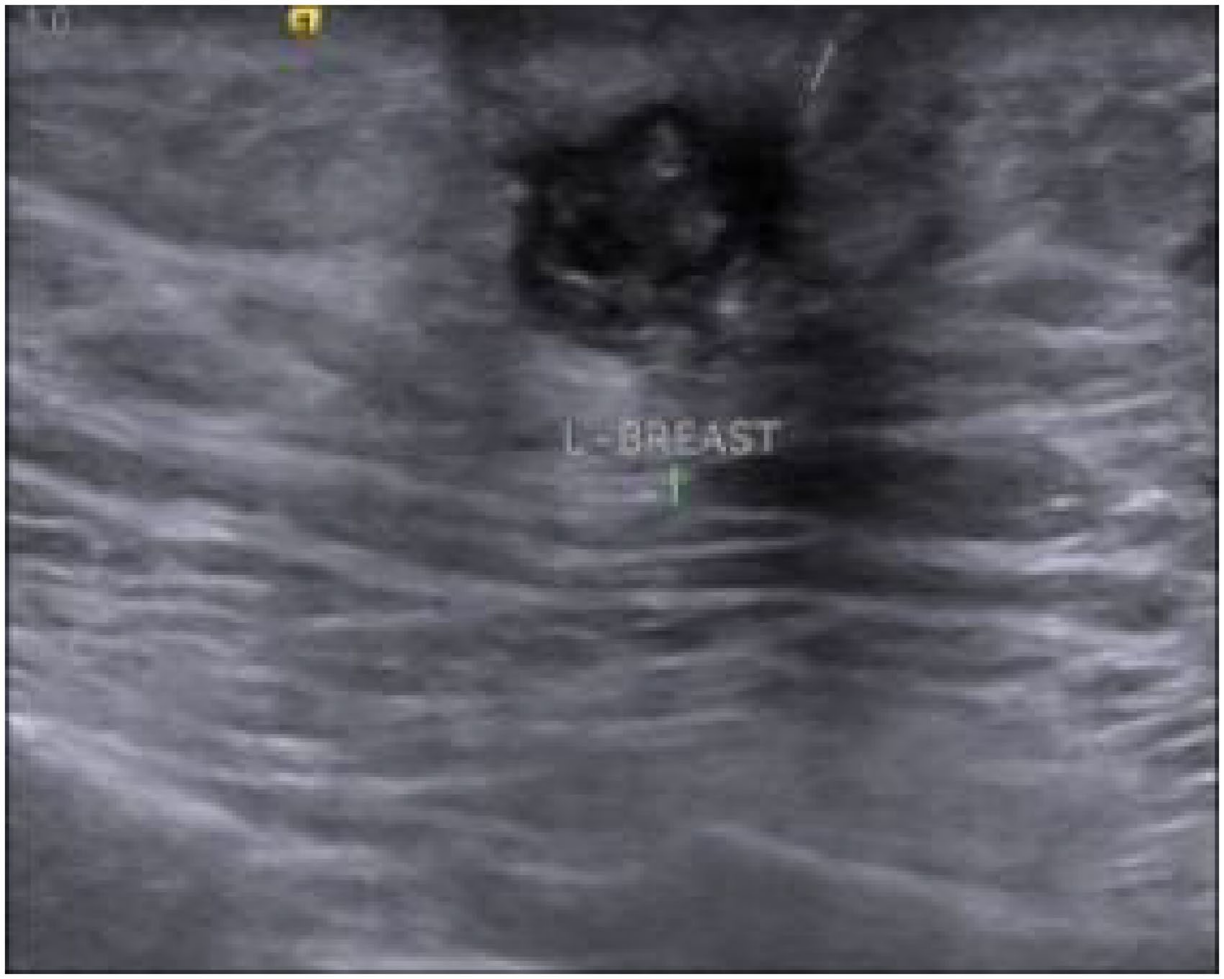
Color Doppler ultrasound of the left breast. A 10 × 8 mm hypoechoic mass at the left nipple root demonstrated irregular spiculated margins (characteristic of malignancy), heterogeneous internal echotexture with focal hyperechogenicity (arrow), and well-defined borders with indistinct posterior acoustic shadowing.

**Figure 2:**
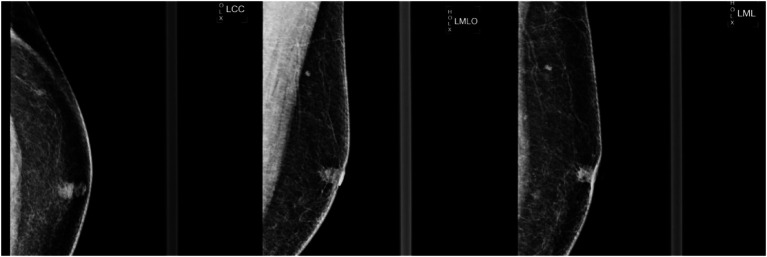
Left breast mammogram in craniocaudal (LCC) mediolateral (LML) and mediolateral oblique (LMO) views. A 10 × 9 mm irregular high-density mass with marked spiculation and architectural distortion was identified in the left subareolar region. The left nipple demonstrated mild inversion (arrow), and no suspicious microcalcifications were noted.

A 55-year-old male (height 170 cm, weight 90 kg, BMI 31 kg/m^2^, nonsmoker) presented to our institution on September 11, 2023, with a 6-month history of a left breast mass. The patient initially self-detected a painless, small subareolar nodule characterized by firm consistency, well-defined margins, and limited mobility, which partially regressed after 1 month of oral amoxicillin (0.5 g twice daily). Subsequent physical examination revealed a fixed inverted left nipple and a 10 × 5 mm indurated subareolar mass with indistinct margins, non-tender to palpation, and no cutaneous adhesion. Bilateral axillary and supraclavicular lymph nodes were unremarkable. Diagnostic imaging demonstrated: (1) ultrasound-detected (Figure 1) hypoechoic left nipple root mass (10 × 8 mm) with irregular spiculated borders, internal calcifications, and heterogeneous echotexture (BI-RADS 4C); (2) mammography-identified (Figure 2) irregular high-density left subareolar mass (10 × 9 mm) with spiculated margins, architectural distortion, and nipple inversion (BI-RADS 5). Laboratory investigations, including complete blood count, serum biochemistry, tumor markers (CEA, CA-153), and hormonal profile (estradiol, FSH), were within normal limits. Given the clinical and radiological features suspicious for malignancy, surgical excision was elected after multidisciplinary discussion despite negative percutaneous biopsy recommendations.

## 3 Operative procedure and pathology

### 3.1 Operative procedure

Under general anesthesia, an excisional biopsy was performed. Intraoperative findings revealed a 1 × 1 cm firm, ill-defined mass at the left subareolar region with surrounding milky secretions. En bloc resection with a 5-mm margin was achieved using electrocautery. Intraoperative frozen section analysis demonstrated chronic inflammation with features suggestive of plasma cell mastitis. The wound was irrigated, and hemostasis confirmed prior to closure with absorbable subcutaneous and intradermal sutures. The patient recovered uneventfully. Twelve-month follow-up confirmed no local recurrence or metastatic disease.

### 3.2 Pathological findings

#### 3.2.1 Gross examination

Gray-yellow irregular tissue (2.1 × 0.8 cm) containing a 0.7 cm gray-tan nodule with circumscribed margins.

#### 3.2.2 Microscopic evaluation

**Figure 3:**
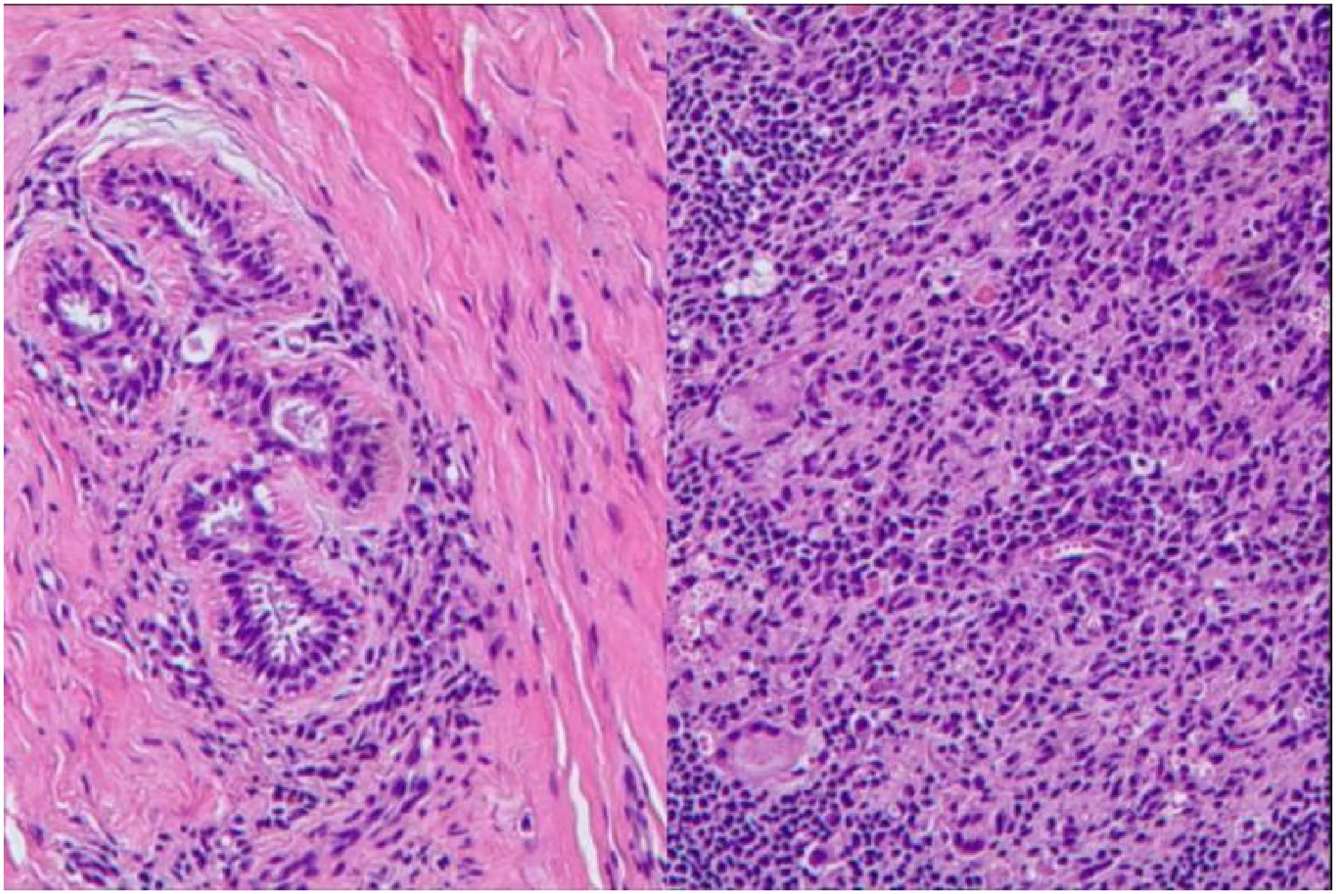
Histopathological examination of the resected breast specimen. Focally extensive infiltration of neutrophils, plasma cells, and multinucleated giant cells (arrows indicate multinucleated giant cells) was observed, consistent with granulomatous inflammation. No malignant cells or atypical hyperplasia were identified.

Focal dense infiltrates of plasma cells, neutrophils, and multinucleated giant cells within breast parenchyma, consistent with plasma cell mastitis (Figure 3).

## 4 Discussion

PCM primarily affects non-lactating women of reproductive age, with a predilection for married multiparous individuals. While male PCM is rare, recent evidence suggests shared and distinct pathogenic mechanisms. Autoimmunity has been implicated as a central driver in both sexes, supported by associations with systemic autoimmune disorders such as diabetes mellitus and leukodystrophy (3). In women, additional risk factors include bacterial infection, nipple inversion, obesity (BMI ≥ 24 kg/m^2^), delayed menarche, early first parity, high gravidity, contraceptive use, hyperprolactinemia, and psychological stress (4).

Male PCM demonstrates unique etiological profiles, including male breast hypertrophy (gynecomastia), smoking, and genetic predisposition (5). In our case, the patient had no history of autoimmune disease, but presented with obesity (BMI 31 kg/m^2^) and nipple inversion—two critical risk factors. Obesity likely contributes via two mechanisms: (1) adipose tissue-secreted adipokines alter estrogen receptor expression in breast parenchyma, disrupting hormonal homeostasis (6); (2) excessive adipose remodeling induces tissue hypoxia, triggering local inflammatory cascades characterized by pro-inflammatory cytokine upregulation (7). For cases where obesity-related adipokine dysregulation and tissue hypoxia perpetuate a persistent chronic pro-inflammatory state, surgical intervention remains the primary therapeutic approach. Concurrently, weight management should be supplemented to address the underlying metabolic driver. Nipple inversion, although less common in males, causes ductal obstruction, keratinocyte debris accumulation, and lipid secretion stasis, creating an antigenic microenvironment that recruits plasma cells and lymphocytes (8, 9).

**Figure 4:**
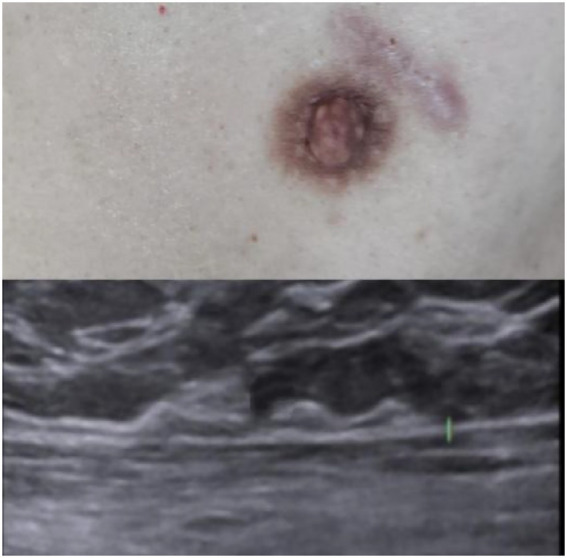
One-year postoperative follow-up of the left breast. Clinical examination revealed a well-healed scar with no infection or dehiscence, symmetric non-inverted nipples, and no palpable masses or lymphadenopathy. Color Doppler ultrasound (12-MHz linear probe) demonstrated homogeneous echotexture in bilateral mammary glands with normal vascularity, no residual lesions, and no focal hyperemia or abnormal color flow signals, consistent with BI-RADS 1 (negative findings).

PCM presents with nonspecific symptoms, including painful subareolar masses, nipple inversion, discharge, and occasional fistulae, while skin erythema, breast hypertrophy, and recurrent abscesses are rare (1). Disease progression is typically staged into secretory, nodular, suppurative, and fistulizing phases (10). Although male PCM follows a similar pathological trajectory to female cases—beginning with ductal dilatation and minimal symptoms before progressing to diffuse solid masses—the clinical presentation in males is often delayed. Palpable breast masses are considered the cardinal feature of male PCM (11) (see Figure 4).

In our case, the patient presented with a long-standing indolent mass, likely due to several factors: (1) absence of acute inflammatory signs (erythema, warmth, tenderness) in early disease; (2) male patients’ reduced breast cancer awareness and reluctance to seek timely medical evaluation; and (3) gradual transition from acute to chronic inflammation, marked by ductal sclerosis and nipple retraction secondary to fibrotic changes. These features frequently mimic breast carcinoma, underscoring the diagnostic challenge in male patients. Ductal obstruction and dilatation constitute the core pathophysiological process of this disease. This process leads to the stasis of secretions and periductal inflammation; therefore, complete duct excision is required to eliminate the inflammatory nidus. Mammography and breast ultrasound lack specificity for non-lactating mastitis (12). Mammograms typically reveal asymmetric focal/diffuse densities, architectural distortion, or ill-defined masses, while ultrasound demonstrates irregular hypoechoic heterogeneous lesions. In our case, both modalities showed a spiculated mass mimicking breast cancer, highlighting the limitations of imaging in distinguishing PCM from malignancy. Histologic analysis remains essential for definitive diagnosis, characterized by dense lymphoplasmacytic infiltrates.

In our review of the literature (Table 1), no standardized treatment exists for PCM. While high-dose glucocorticoids (0.5–1 mg/kg/d) may transiently control inflammation, relapse commonly occurs upon dose reduction (9). Methotrexate has shown efficacy in idiopathic granulomatous mastitis (13). For patients with evidence of autoimmune dysregulation who present with diffuse or recurrent lesions, immunosuppressants—including corticosteroids and methotrexate—can control systemic immune activation. Surgical intervention is preferred due to high recurrence rates, with options including ultrasound-guided drainage, excision, or mastectomy. Lesionectomy suffices for localized disease (12), while total mastectomy is reserved for cases indistinguishable from carcinoma (14). For recurrent abscesses/fistulae, treatment depends on ductal involvement: fistulotomy for single-duct disease vs. complete duct excision for multifocal involvement (11).

**Table 1:** Information on male patients with plasma cell mastitis.

Age	Clinical manifestations	Treatment methods	Prognosis
38 years old	1-month history of a palpable parapillary mass, erythema, and localized pain on the right chest	Antibiotics + Prednisone + Minimally invasive rotational resection + Right mastectomy (15)	Recurrence
51 years old	Erythema and pain in the right breast for 8 days	Needle biopsy + Rupi Sanjie Granules (a traditional Chinese medicine granule for relieving breast lumps and dissipating nodules) (16)	Stable condition
30 years old	Recurrent mass with erythema and pain in the right breast for more than 2 years	Needle aspiration + Traditional Chinese medicine + Fistula resection (17)	Stable condition
45 years old	Swelling and pain in the left breast for 10 days	Needle aspiration + Traditional Chinese medicine + Incision and drainage of abscess (18)	Stable condition
57 years old	Mass in the left breast for 3 years, complicated with erythema and pain for 1 week	Tamoxifen + Needle aspiration + Oral and external application of traditional Chinese medicine (19)	Stable condition

In our patient, self-administered antibiotics initially masked infection, leading to a 1-cm indolent mass at presentation. Lumpectomy was performed after shared decision-making, resulting in complete resolution and no recurrence at 12-month follow-up.

## 5 Conclusion

Male breast masses should prompt clinical suspicion of plasma cell mastitis (PCM), with definitive diagnosis requiring pathological confirmation. Based on this case, timely and appropriate surgical intervention for male PCM in the nodular phase can significantly improve outcomes and minimize recurrence risk. However, the role of adjuvant therapies (e.g., glucocorticoids) in preventing relapse remains unclear and warrants further investigation. Prospective studies are needed to establish evidence-based guidelines for optimal management strategies in male patients.

## Data Availability

The raw data supporting the conclusions of this article will be made available by the authors, without undue reservation.
